# HOMA-IR index in non-diabetic patient, a reliable method for early diagnosis of liver steatosis

**DOI:** 10.22088/cjim.13.3.519

**Published:** 2022

**Authors:** Behrang Motamed, Mahsa Kohansal Vajargah, Saeed Kalantari, Afshin Shafaghi

**Affiliations:** 1 Department of Internal Medicine, Razi Hospital, School of Medicine, Guilan University of Medical Sciences, Rasht, Iran; 2 Razi Clinical Research Development Unit, Guilan University of Medical Sciences, Rasht, Iran; 3 GI Cancer Screening and Prevention Research Center, Guilan University of Medical Sciences, Rasht, Iran

**Keywords:** NAFLD, HOMA-IR, insulin resistance, FibroScan, BMI, non-diabetic

## Abstract

**Background:**

NAFLD is one of the most common liver diseases in the world. HOMA-IR as an indicator of insulin resistance is commonly used in clinical trials in NAFLD patients. The aim of this study was to evaluate the application of HOMA-IR index in the diagnosis of NAFLD.

**Methods:**

This study was performed on 54 patients with NAFLD and 54 non-NAFLD patients that referred to Razi Hospital in Rasht during 2019-2020. FibroScan was used to diagnose NAFLD in the patient group and ultrasound was used to rule it out in the control group. Metabolic and hepatic parameters were measured for each patient. Data were entered into SPSS 22 software and the necessary analyses were performed.

**Results:**

The mean age of the subjects in the study was 44.01±13.12 years and ranged from 18 to 75 years. 72.2% of people affected by NAFLD were men (p <0.001) .The optimal cut-off point for HOMA-IR in NAFLD was 1.65 with a sensitivity of 89.7% and a specificity of 76.9% in men and 1.90 with a sensitivity of 86.7% and a specificity of 82.9% in women. Overall, the optimal cut-off point for HOMA-IR in NAFLD was 1.75 with a sensitivity of 87.0% and a specificity of 81.5%. In addition, the results showed that there was no significant relationship between steatosis and hepatic fibrosis with HOMA-IR index.

**Conclusion:**

The results showed that HOMA-IR can be used as a reliable criterion for early detection of NAFLD.

Non-alcoholic fatty liver disease (NAFLD) is one of the most common liver diseases in many parts of the world. This disease is known as one of the most important causes of mortality due to liver disorders (1, 2). Non-alcoholic fatty liver disease can lead to cirrhosis and liver failure. NAFLD is defined as the accumulation of fat in the liver in not consuming too much alcohol. The disease is caused by the accumulation of triglycerides and other fats in liver cells (3-6). Hepatic steatosis accounts for approximately 25 to 35% of the population (7). NAFLD is currently one of the most common causes of chronic liver disease in young people in both developed and developing countries (8-10). The prevalence of non-alcoholic fatty liver disease is increasing in parallel with the prevalence of obesity and its rate is reported as 54.4% in Iran. It is also stated that non-alcoholic fatty liver disease is closely related to metabolic diseases such as type 2 diabetes and obesity that both have insulin resistance. Some studies have also shown that NAFLD can increase the risk of developing diabetes (11-13). Evaluation of Homeostatic Model Assessment for Insulin Resistance (HOMA-IR) has become the most commonly used method in both clinical application and epidemiological studies (14, 15).

HOMA-IR was proposed by Matthews et al. (16) and found to have a significant correlation with glucose control in non-diathetic patients and is commonly used in clinical trials in NAFLD patients (17, 18). The aim of this study was to evaluate the diagnostic value of HOMA-IR to identify patients with NAFLD confirmed by FibroScan and determine the optimal cut-off point for it. In the present study, HOMA-IR can be used as a method in the early diagnosis of NAFLD disease by determining the optimal cut-off point of HOMA-IR to identify the patients with NAFLD and obtaining the desired results.

## Method

This study was performed on 108 patients (54 patients with NAFLD and 54 healthy individuals) referred to Razi Hospital in Rasht in 2019-2020 who were selected in the available sampling method for inclusion in the study. The plan was implemented after reviewing and receiving the code of ethics in the Ethics Committee of Guilan University of Medical Sciences based on the criteria of the 31 principles of ethics (IR.GUMS.REC.1398.498) and obtaining informed written consent after providing sufficient explanations about the plan to all patients.

An information form was completed for each patient, including background information (age, sex, place of residence, BMI, waist circumference) and clinical examination. Patients for whom the NAFLD diagnosis was approved based on guidelines (confirmed using fibroscan) were included in the study as the affected group. The non- affected group was selected from healthy individuals referring to the endocrinology clinic of Razi Hospital in Rasht who were eligible for inclusion in the study and had normal sonography. The CAP value was also calculated using the results of fibroscan. In this study, the fibroscan diagnostic system was used. The model of this system is Fibro Touch-FT100 with Power: 12V --- 10A(Shanghai International Holding Corp GmbH(Europe) Hamburg, Germany) 

After obtaining patients' consent, fasting blood samples were taken from them (8 hours) and fasting insulin indices FBS, ALT, AST, ALK.P were measured for each patient. All experiments were measured in private and unit laboratories using unit kits. Fasting blood sugar and fasting insulin were used to measure insulin resistance according to the following equation

HOMA-IR = [glucose (nmol.L) * insulin (µU.mL).22.5]

Fasting blood glucose was measured by Bionik kit by Glucose oxidase method, fasting blood insulin level was measured by Diasorin Liasion kit by CLIA method and AST, ALT, Alk.Pho tests were measured by Bionik kit by Enzyme kinetics method. In this study, data were collected, coded and entered into SPSS 22 software. Mean and standard deviation (95% confidence interval) were used to describe quantitative variables with normal distribution and median and interquartile range were used for quantitative variables with abnormal distribution. The normal distribution of the study variables was measured using the Shapiro – Wilk test. 

Comparison of demographic and clinical characteristics in the two groups of non-alcoholic fatty liver and healthy individuals was performed using independent t-test or its non-parametric equivalent Mann-Whitney, chi square and Fisher's exact test. The correlation of CAP value with HOMA-IR was calculated using Pearson (or Spearman) correlation coefficient test. The best cut-off point for HOMA-IR in the diagnosis of non-alcoholic fatty liver was calculated using the ROC curve. The level of statistical significance of the tests was considered p<0.05.

## Results

In this study, 108 people were included, of which 54 were non-alcoholic fatty liver patients and 54 were healthy individuals.The results showed that the mean age of participants in the study was 44.01±13.12 years with a median of 41.00 and an age range of 18 to 75 years. The age group of non-alcoholic fatty liver patients was older than non-alcoholic individuals, but this difference was not statistically significant (p=0.169). 

There was a statistically significant difference between the two groups in terms of gender distribution (p <0.001) hence, 72.2% of patients with non-alcoholic fatty liver were men and 27.8% were women. Based on the results of body mass index status between the two groups, a statistically significant difference was observed between the groups with and without non-alcoholic fatty liver (p<0.001). The demographic information of the patients is given in table 1. Experimental data showed that fasting insulin levels (p<0.001), AST (P=0.042) and ALT (P=0.005) in the group with non-alcoholic fatty liver were significantly higher than the non-alcoholic group (table 2).

**Table 1:** Demographic characteristics of the two groups with non-alcoholic fatty liver and healthy individuals

**Variable**		**Group with non-alcoholic fatty liver** ** (N=54)**	**Healthy individuals (N=54)**	**P-value**
Age (years), mean ± standard deviation, mean (min-max)		45.74±12.71 (23.00-72.00) 42.00	42.28土13.41 (18.00-75.00) 40.50	0.169*
Gender, number (percentage)	Male	39 (72.2)	13 (24.1)	0.001**
	female	15 (27.8)	41 (75.9)	
BMI (kg.m2) mean ± standard deviation, mean (min-max)		28.44土5.63 (21.00-43.90) 27.75	21.21土2.44 (18.00-28.00) 20.50	0.001*
BMI (kg.m2) number (percentage)	< 18.5	0	2 (3.7)	0.001***
	18.5-24.9	20 (37.0)	47 (87.0)	
	25.0-29.9	15 (27.8)	5 (9.3)	
	˃30	19 (35.2)	0	
Waist mean ± standard deviation, mean (min-max)		112.15土4.12 -120.00 111.50 (101.00	105.68土8.06 -119.00 106.00 (76.00	0.001*

**Table 2:** Comparison of laboratory factors in non-alcoholic fatty liver and healthy individuals

variable	Group with non-alcoholic fatty liver (n=54)	Healthy individuals (n=54)	P-value
**Fasting insulin levels**	14.04±5.33 13.10 (4.80-24.00)	6.43±4.23 4.50 (3.10-22.50)	<0.001*
**FBS (kg.m2)**	89.78±5.62 90.00(76.00-99.00)	88.04±5.98 90.00(77.00-98.00)	0.216*
**AST (U.L) **	23.20±9.04 21.00(10.00-51.00)	19.80±7.22 19.00(10.00-38.00)	0.042*
**ALT (U.L)**	30.80±21.33 21.50(7.00-90.00)	19.94±8.48 17.50(10.00-42.00)	0.005*
**ALKP (U.L) **	153.56±45.71 137.50 (89.00-290.00)	161.09±44.25 152.00(86.00-274.00)	0.182*

**Figure 1:**
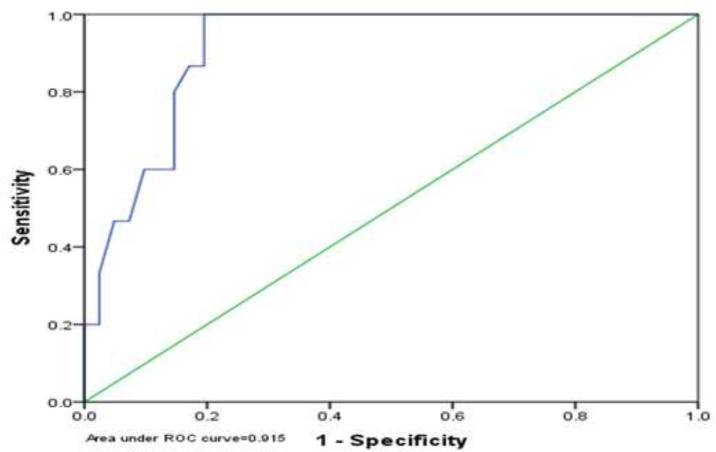
HOMA-IR cutting point in NAFLD in men

**Figure 2:**
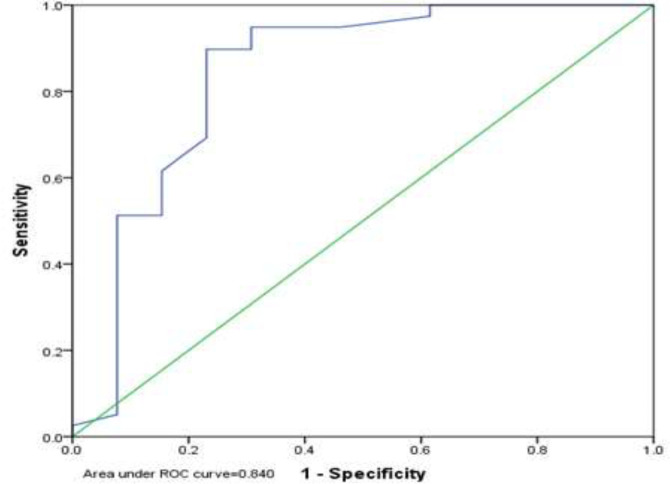
HOMA-IR cutting point in NAFLD in women

**Figure 3:**
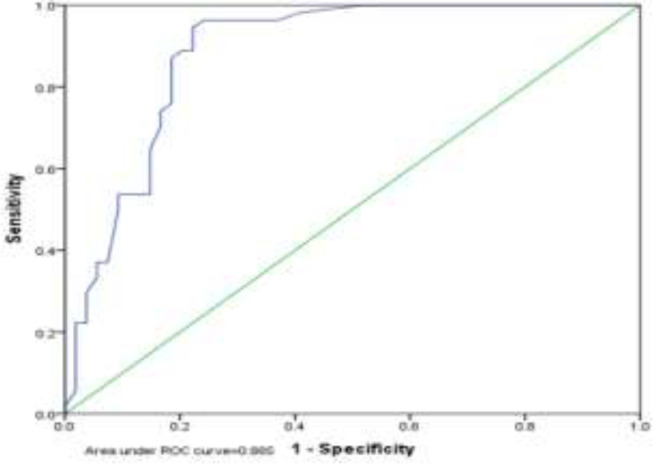


In addition, the Homeostatic Model Assessment for Insulin Resistance (HOMA-IR) in the group with non-alcoholic fatty liver was significantly higher than the non-alcoholic group (p <0.001) (table 3). The optimal cut-off point for HOMA-IR in NAFLD was 1.65 with a sensitivity of 89.7% and a specificity of 76.9% in men and 1.90 with a sensitivity of 86.7% and a specificity of 82.9% in women. In general, the best positive point for the mentioned index was 1.75 with a sensitivity of 87.0% and a specificity of 81.5% (figures 1, 2, 3). In addition, the results showed that there was no significant relationship between steatosis and hepatic fibrosis with HOMA-IR index (table 4).

**Table 3:** Comparison of homeostatic index of insulin resistance (HOMA-IR) in patients with non-alcoholic fatty liver disease and healthy individuals

**Variable**	**Group with non-alcoholic fatty liver** ** (n=54)**	**Healthy individuals (n=54)**	**P-value**
**HOMA-IR **	3.15±1.29 3.15 (1.00-7.20)	1.42±1.01 1.00 (0.70-5.40)	> 0.001*

**Table 4:** Correlation of steatosis and hepatic fibrosis with hemostatic index of insulin resistance (HOMA-IR) in patients with non-alcoholic fatty liver

**Variable**	**Type of correlation coefficient**	**The value of the correlation coefficient**	**P- value**	**Type of correlation**
Hepatic steatosis (based on UAP)(dB . m)	Spearman	0.159	0.251	Very weak insignificant correlation
Hepatic fibrosis (based on KPA)	Spearman	0.100	0.472	Very weak insignificant correlation

. HOMA-IR cutting point in NAFLD in both sexes.

## Discussion

Many tests are used to assess insulin resistance, such as Quicki test, insulin sensitivity index, etc., but the HOMA-IR index is most used in daily visits and epidemiological studies (19). Insulin resistance appears to be more common in patients with nonalcoholic fatty liver disease (20). In the present study, the mean age of people in the non-alcoholic fatty liver group was higher than healthy individuals. However, this difference was not statistically significant. This may be due to the small number of samples in this study or the effects of racial and genetic factors on this issue. Non-alcoholic fatty liver disease occurs at all ages. However, aging is one of the risk factors for NAFLD, which can be due to the effects of factors such as chronic diseases, inactivity and obesity (21, 22).

There was a statistically significant difference between the two groups in terms of gender distribution, thus 72.2% of patients were men. Although fatty liver has previously been reported more often in women, recent studies have reported a higher incidence in men. Recent demographic studies in this regard have led to the contradictory results (23). In a study of adults in Shanghai, only 10 percent of fatty liver patients were women (24). Another clinicopathological study in India considers the sexual preference of non-alcoholic fatty liver disease in men (25). On the other hand, there are other studies that consider the prevalence of non-alcoholic fatty liver disease in women (26). Likewise, in this study, they confirm a significant association between NAFLD and obesity as well as an increase in BMI (27-29). Moreover, waist circumference of patients with non-alcoholic fatty liver was significantly higher than the healthy individuals. Obesity is one of the most important diseases associated with fatty liver. However, increasing the amount of abdominal fat compared to obesity is a more important indicator of non-alcoholic fatty liver disease (30). In the present study, FBS and fasting insulin levels were used to evaluate the indicators related to diabetes, which showed a statistically significant difference between them and NAFLD. Insulin resistance, which is a precursor to diabetes, is the basis of metabolic syndrome and NAFLD and can adversely affect liver cells even before the onset of overt diabetes (31).

The results of this study showed that the homeostatic index of insulin resistance (HOMA-IR) in the group with non-alcoholic fatty liver was significantly higher than the healthy group. This difference was reported to be statistically significant, consistent with the study by Villanueva, 2019 (32), Al Hossain et al., 2016 (33), Gruben et al., 2014 (34) and Pirgon 2013 (35). Their results showed that NAFLD patients had higher levels of insulin, glycemia and HOMA-IR compared to the control group. Furthermore, the optimal cut-off point for HOMA-IR in NAFLD was 1.65 with a sensitivity of 89.7% and a specificity of 76.9% in men and 1.90 with a sensitivity of 86.7% and a specificity of 82.9% in women. In general, the best positive point for the mentioned index was 1.75 with a sensitivity of 87.0% and a specificity of 81.5%.

In a 2010 study by Salgado et al., A HOMA-IR index equal to or greater than 2 or 2.5 resulted in an increase in diagnostic value in patients with NAFLD compared with controls (36). Besides, the study of Motamed et al. in 2016 showed that the optimal cut-off point for HOMA-IR in NAFLD was 1.79 with a sensitivity of 66.2% and a specificity of 62.2% in men and 1.95 with a sensitivity of 65.1% and a specificity of 54.7% in men (37).

In a 2017 study by Guttirez-Buey et al., the best cut-off point obtained to differentiate NAFLD from non-NAFLD was estimated to be 4.5 (38). In a 2017 study by Isokuortti et al., the cut-off point of the HOMA-IR index for NAFLD was 1.9 (with a sensitivity of 87% and a specificity of 79%) (39). 

According to these results, the cut-off point determined in our study is lower than all studies. The reason for this difference could be the use of fibroscan in this study, which detects fatty liver in the early stages and is more efficient than ultrasound, which has been used as a diagnostic criterion in other studies (40).

In addition, the results of the present study indicated that there was a very weak positive (direct) non-significant correlation between severity steatosis and hepatic fibrosis with HOMA-IR index, although low sample size could be a reason for not finding this relationship; and in the case of more samples, maybe the different results would be obtained. On the other hand, considering that the HOMA-IR index shows the degree of insulin resistance, and in addition to insulin resistance, fat accumulation also plays a role in NAFLD, they can explain this very weak correlation (41) In this way, besides this method CAR-T cell therapy or stem cell therapy recommended in liver diseases (42, 43) also molecular test to investigate their cellular mechanisms is recommended (44-47). In general, the results of this study showed that HOMA-IR can be used as a laboratory computational index with an optimal cut-off point of 1.75 in the diagnosis of NAFLD in non-diabetic patients. It is suggested that this index be used as a method for selecting and referring patients for further evaluation, due to its availability and low cost. Moreover, to achieve a more accurate cut-off point in future studies, a higher sample size should be used; and according to the influence of racial and genetic factors, this index should be calculated in separate populations for each specific geographical area.
